# The importance of better natural history studies for Duchenne muscular dystrophy

**DOI:** 10.1111/dmcn.16306

**Published:** 2025-03-18

**Authors:** David J. Birnkrant

**Affiliations:** ^1^ Case Western Reserve University School of Medicine – Pediatrics Cleveland Ohio USA

## Abstract

This commentary is on the original article by Chesshyre et al. on pages 1280–1289 of this issue.

Duchenne muscular dystrophy (DMD)^1^ has entered an era of molecular and genetic therapies.^2^ The study of those therapies relies heavily on natural history data, which are used for historical controls.

The study by Chesshyre et al.^3^ aims to improve the accuracy of that natural history data. DMD is subject to phenotypic variability by which individuals with the same dystrophin mutation can have very different muscle strength and cardiorespiratory function, due to genetic modifiers.^2^, ^4^ One such modifier is isoform Dp140 and the study reports that pinch and grip strength, and one pulmonary function outcome, were statistically worse in the group with Dp140 disruption.^3^ The individuals were a mixed group, from three different natural history studies.

Would matching individuals for Dp140 deficiency make natural history studies more accurate? Should drug studies also match controls and treated individuals for Dp140 status?

First, consider that Dp140 is just one DMD modifier and it may not be the most impactful one. Overall, the identification of all the DMD modifiers, and their relative clinical importance, remains investigational.^2^, ^4^ Moreover, phenotypic variability due to genetic modifiers is just one example of the uncontrolled variables affecting natural history studies, including this one. Recently, we classified phenotypic variability as an uncontrolled intrinsic variable.^5^ Uncontrolled extrinsic variables include variations in standards of care, such as treatment with glucocorticoids, which benefit muscle strength and pulmonary function.^1^ In the study by Chesshyre et al.,^3^ individuals are categorized as glucocorticoids ‘exposed’ or ‘naive’. There are no details on glucocorticoids formulation, schedule, dosing, or adherence – all of which can affect strength and pulmonary function. Also, pulmonary function measurements were ‘… collected using different equipment at different sites and there wasn't centralized monitoring of the technique …’^3^ This is an uncontrolled technical variable. Forced vital capacity (FVC) is a highly standardized maneuver, with strict criteria for validation. Without those standards, individual variations in skill and effort are quite likely to affect the results.

Some combination of these uncontrolled variables might explain why, even after sorting by Dp140 expression, the individual FVC percentage predicted (% pred) results shown in Figure 2 vary widely.^3^ That kind of variability is common in natural history studies. As in Figure 2, the scatter is smoothed using statistical techniques and the respiratory results are reported as a single constant rate of decline. However, that constant rate of decline contains a crucial flaw – it masks the underlying heterogeneity of the actual data.^5^ Most DMD drug studies compare a single rate of decline in FVC % pred between historical controls and treated individuals. This methodology may be flawed, making assessments of drug effectiveness potentially inaccurate.^5^

Here is another consideration: DMD's major complications are determined by cardiorespiratory function – which does not fall to clinically impactful levels until individuals are ‘older’ (i.e. in the late, non‐ambulatory stage of the disease).^5^ In the current study, the mean age at first visit of the respiratory group is 8 years 6 months and mean age at last visit is 11 years 5 months (Table 1).^3^ These young individuals are likely ambulatory – a group that essentially never experiences cardiorespiratory morbidities. Thus, even if Dp140 disruption causes deficits in hand strength and FVC % pred that are statistically significant, the clinical significance of those results can be questioned.^5^

These limitations, which are shared by most DMD natural history studies, are why new natural history studies are needed that are both prospective in nature and designed to minimize the effect of uncontrolled variables. The emphasis should be on cardiorespiratory outcomes, which are the determinants of survival, with an adequate number of individuals who are ‘older’ and non‐ambulatory.

Focusing on more accurate natural history data could improve the design of future drug studies; and better drug studies could bring better therapies to individuals with DMD.^5^

## Data Availability

Not required.
